# Prevalence and related factors of depressive symptoms among HIV/AIDS in Ningbo, China: A cross-sectional study

**DOI:** 10.3389/fpsyt.2022.1004318

**Published:** 2022-10-10

**Authors:** Suting Chen, Hang Hong, Guozhang Xu

**Affiliations:** ^1^School of Medicine, Ningbo University, Ningbo, China; ^2^Ningbo Municipal Center for Disease Control and Prevention, Ningbo, China

**Keywords:** HIV, antiretroviral therapy, depressive symptoms, prevalence, related factors

## Abstract

**Background:**

Depressive symptoms were common among HIV/AIDS patients. Previous studies had shown that HIV-infected patients were twice as likely to be diagnosed with depression as the general population. However, only few studies have explored the prevalence and related factors of depressive symptoms among HIV/AIDS in China.

**Materials and methods:**

A cross-sectional study was conducted to study the prevalence of depressive symptoms among HIV/AIDS from January to December 2021 through the database of HIV/AIDS antiretroviral therapy and psychological evaluation system in Ningbo, China. The Patient Health Questionnaire-2 (PHQ-2) was used to screen for depressive symptoms (PHQ-2 > 0), the Patient Health Questionnaire-9 (PHQ-9) was used to diagnose depressive symptoms, and multivariate *Logistic* regression model was carried on to evaluate the related factors.

**Results:**

A total of 3,939 HIV/AIDS patients were enrolled, and the age of initiation of antiretroviral therapy was 37.15 (IQR = 28.41–48.73) years. Among them, 3,230 (82.00%) were male, 3,844 (97.59%) were Han nationality, 1,391 (35.49%) were unmarried, 1,665 (42.27%) were homosexual transmission, and 2,194 (55.70%) were HIV-infected patients. There were 265 patients (6.73%) with depressive symptoms, and the proportion of mild, moderate, moderate and severe depressive symptoms was 4.01% (158/3939), 1.65% (65/3939), 0.76% (30/3939), and 0.30% (12/3939), respectively. Multivariate analysis showed that married [odds ratio (OR) = 0.675, 95% CI = 0.501–0.908], divorced or widowed (OR = 0.571, 95% CI = 0.380–0.860), homosexual transmission (OR = 1.793, 95% CI = 1.349–2.396) were associated with depressive symptoms among HIV/AIDS.

**Conclusion:**

The prevalence of depressive symptoms among HIV/AIDS patients was 6.73% in Ningbo, China. More attention should be paid to the psychological status of unmarried and homosexual HIV/AIDS patients in Ningbo and timely psychological intervention or treatment should be given to those patients with depressive symptoms.

## Introduction

The widespread application of antiretroviral therapy has greatly reduced the morbidity and mortality of HIV/AIDS patients, thus realizing the goals of HIV suppression and prolonging the life of patients (1, 2). The improvement of life quality among HIV/AIDS was accompanied by the onset of some psychiatric symptoms such as depression (3–5). It was estimated that depression alone may be one of the three leading causes of disease burden in low-income countries by 2030 (6, 7). Therefore, preventing the incidence of depression is extremely important among HIV/AIDS patients.

The incidence of depressive symptoms was higher among HIV-infected patients (8–11). Previous studies have shown that people living with HIV were twice as likely as the general population to be diagnosed with depression (10). Depression is a debilitating condition that adversely affects adherence to antiretroviral therapy and viral suppression among HIV/AIDS (12–15), thereby reducing the life quality of patients (16). It has been found that the 2-year mortality risk of those with depressive symptoms was 9.7%, higher than that of those without depressive symptoms among HIV-infected patients who inject drugs (17). Recent systematic reviews about the relationship between HIV and depressive symptoms showed that depressive symptoms were associated with gender, age, marital status, economic level, social support, HIV-1 RNA level, CD4 count, antiviral therapy, sexual transmission, opportunistic infections and social stigma among HIV/AIDS (18–22).

The prevalence and related factors of depressive symptoms among HIV/AIDS have been rarely studied in China; yet there is a need to investigate the related factors of depressive symptoms among HIV/AIDS. Therefore, in order to understand the depressive symptoms and related factors in HIV/AIDS patients, further improve the compliance of antiretroviral therapy and life quality of patients, this study conducted a cross-sectional study among HIV/AIDS patients who had been treated with antiretroviral therapy in Ningbo, China.

## Materials and methods

### Participants

The HIV/AIDS patients in Ningbo before December 31, 2020 were selected. The inclusion and exclusion criteria were as follows. Inclusion criteria: (1) informed consent; (2) treatment status of HIV/AIDS was “new treatment,” “under treatment,” or “referral” and the card review mark was “final approval card”; (3) age ≥ 18 years; (4) psychological evaluation was conducted and the results were complete; (5) complete recent viral load information. Exclusion criteria: (1) informed consent was not obtained; (2) subjects of lost follow-up, death or long-term absence; (3) age < 18 years; (4) without psychological evaluation; (5) without recent viral load information.

### Measures

**FIGURE 1:**
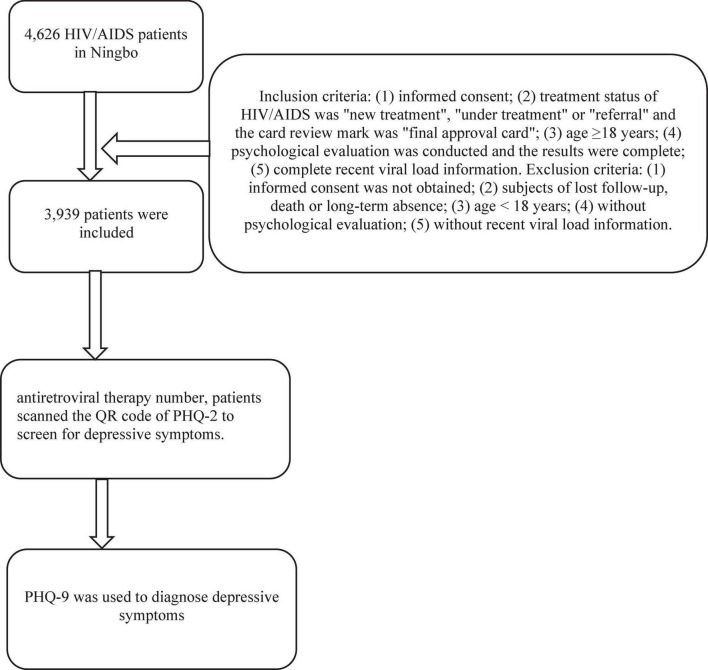
Flowchart for the data collection.

Based on the database of HIV/AIDS antiretroviral therapy and the psychological evaluation network system of patients, the general demographic information, basic information of antiretroviral therapy and psychological evaluation information were collected from January to December 2021. The information includes: (1) general demographic information: gender, age, education, marital status, occupation, etc. (2) basic information of antiretroviral therapy: sexual transmission, WHO clinical stage, number of disease symptoms at baseline, positive date of HIV antibody test, start date of antiretroviral therapy, antiretroviral drugs, number of baseline CD4 + T lymphocytes (CD4 count for short), etc. (3) psychological evaluation information: The assessment of depressive symptoms was a two-stage screening. Follow-up doctors in Voluntary Counseling and Testing (VCT) outpatient initially provided antiretroviral therapy number, patients scanned the QR code of The Patient Health Questionnaire-2 (PHQ-2) (23) to screen for depressive symptoms. The Patient Health Questionnaire-9 (PHQ-9) (24) was used to diagnose depressive symptoms secondly if PHQ-2 scores higher than zero (Figure 1).

The PHQ-2 (23) scale consisted of two items and was rated from 0 (none at all) to 3 (almost daily). The contents include: (1) little interest or pleasure in doing things, (2) feeling down, depressed, or hopeless. There were nine items in the PHQ-9 (24) scale, and the score was 0 (not at all) to 3 (almost every day). These include: (1) little interest or pleasure in doing things, (2) feeling down, depressed, or hopeless, (3) trouble falling or staying asleep, or sleep too much, (4) feeling tired or having little energy, (5) poor appetite or overeating, (6) feeling bad about yourself-or that you are a failure or have let yourself or your family down, (7) trouble concentrating on things, such as reading the newspaper or watching television, (8) moving or speaking so slowly that other people could have noticed? Or the opposite-being so fidgety or restless that you have been moving around o lot more than usual, and (9) thoughts that you would be better off dead or hurting yourselves in some way.

### Definition of related indicators

The criteria for timely treatment was calculated by referring to the positive date of HIV antibody test and the start date of antiretroviral therapy, and the time interval between two dates ≤ 30 days was defined as timely treatment.

Participants with PHQ-9 scores higher than four was defined as patients with depressive symptoms in the second stage of psychological assessment (24). The PHQ-9 scale scores ranged from 5 to 9 as mild depressive symptoms, from 10 to 14 as moderate depressive symptoms, from 15 to 19 as moderate to severe depressive symptoms, and from 20 to 27 as severe depressive symptoms (24).

### Statistical analysis

Statistical analysis was conducted using the Statistical Package for the Social Science (SPSS), version 26.0. Continuous data were expressed by geometric mean ± standard deviation, and differences between the depressive and non-depressive groups were examined by a completely randomized two-sample *t-test* or *Wilcoxon rank-sum test*. Categorical data were summarized by proportions, and differences between groups were examined by χ^2^ test. Multiple *Logistic* regression model (*Forward: LR*) was used to analyze the related factors of depressive symptoms, and *P* < 0.05 was considered statistically significant.

## Results

### General characteristic of participants

There were 4,626 HIV/AIDS patients in Ningbo before December 31, 2020, and 3,939 patients met the inclusion and exclusion criteria. Among 3,939 subjects, the initial age of antiretroviral therapy was 37.15 (IQR = 28.41–48.73) years. Of 3,939 participants, 3,230 were males (82.00%), 3,844 were Han nationality (97.59%), 1,391 were unmarried (35.49%), 1,367 had junior middle school education level (34.70%), 1,665 were homosexual transmission (42.27%). The time interval from HIV antibody positive to antiretroviral therapy ≤30 days accounted for 60.85%, and the primary treatment regimen was TDF + 3TC + EFV (56.79%) (TDF, tenofovir; 3TC, lamivudine; EFV, efavirenz). Among 3,939 HIV/AIDS patients, 2,194 were HIV infection (55.70%), and 1,745 were AIDS patients (44.30%). There were statistically significant differences between HIV infected patients and AIDS in terms of gender, age at which antiretroviral therapy started, education, marital status, occupation, household registration, WHO clinical stage, sexual transmission and initial treatment regime. Details see Table 1.

**TABLE 1:** Socio-demographic and related characteristics of participants.

	Total (*N* = 3,939)	HIV-infected patients (*n* = 2,194)	AIDS (n = 1,745)	χ ^2^ value	*P-value*
Year of inclusion				184.431	<0.001
2,005–2,009	106 (2.69)	4 (3.77)	102 (96.23)		
2,010–2,012	404 (10.26)	163 (40.35)	241 (59.65)		
2,013–2,015	1,030 (26.15)	560 (54.37)	470 (45.63)		
2,016–2,018	1,425 (36.18)	864 (60.63)	561 (39.37)		
2,019–2,020	974 (24.73)	603 (61.91)	371 (38.09)		
Sex				4.325	0.038
Female	709 (18.00)	370 (52.19)	339 (47.81)		
Male	3,230 (82.00)	1,824 (56.47)	1,406 (43.53)		
Initial age of antiretroviral therapy (years)				134.037	<0.001
≤29	1,142 (28.99)	791 (69.26)	351 (30.74)		
30–44	1,485 (37.70)	792 (53.33)	693 (46.67)		
45–59	906 (23.00)	431 (47.57)	475 (52.43)		
≥60	406 (10.31)	180 (44.33)	226 (55.67)		
Nationality				1.131	0.288
Ethnic minorities	95 (2.41)	58 (61.05)	37 (38.95)		
Han nationality	3,844 (97.59)	2,136 (55.57)	1,708 (44.43)		
Education				69.291	<0.001
Primary and below	817 (20.74)	374 (45.78)	443 (54.22)		
Junior high school	1,367 (34.70)	735 (53.77)	632 (46.23)		
High school or technical secondary school	840 (21.33)	490 (58.33)	350 (41.67)		
Junior college or above	915 (23.23)	595 (65.03)	320 (34.97)		
Marital Status^#^				78.175	<0.001
Unmarried	1,391 (35.49)	905(65.06)	486 (34.94)		
Married	1,861(47.49)	938(50.40)	923(49.60)		
Divorced or widowed	667(17.02)	336(50.37)	331(49.63)		
Occupation*				50.475	<0.001
Workers and migrant workers	656(16.68)	357(54.42)	299(45.58)		
Housekeeping, housework, and unemployment	709(18.03)	402(56.70)	307(43.30)		
Farmers	845(21.48)	397(46.98)	448(53.02)		
Business services	749(19.04)	483(64.49)	266(35.51)		
Others	974(24.76)	551(56.57)	423(43.43)		
Household registration				35.398	<0.001
Ningbo	2,364(60.02)	1,226(51.86)	1,138(48.14)		
Other parts of Zhejiang Province	221(5.61)	138(62.44)	83(37.56)		
Other provinces	1,354(34.37)	830(61.30)	524(38.70)		
WHO clinical stages				455.529	<0.001
Phase I	2,706(68.70)	1,648(60.90)	1,058(39.10)		
Phase II	681(17.29)	462(67.84)	219(32.16)		
Phase III	331(8.40)	75(22.66)	256(77.34)		
Phase IV	221(5.61)	9(4.07)	212(95.93)		
Sexual transmission				91.583	<0.001
Heterosexual	2,082(52.86)	1,026(49.28)	1,056(50.72)		
Homosexual	1,665(42.27)	1,074(64.50)	591(35.50)		
Others	61(1.55)	33(54.10)	28(45.90)		
Unknown	131(3.33)	61(46.56)	70(53.44)		
Opportunistic infections at baseline				174.713	<0.001
No	3,733(94.77)	2,171(58.16)	1,562(41.84)		
Yes	206(5.23)	23(11.17)	183(88.83)		
Duration of antiretroviral therapy (year)				152.834	<0.001
≤3	1,247(31.66)	758(60.79)	489(39.21)		
4–6	1,362(34.58)	837(61.45)	525(38.55)		
7–9	912(23.15)	476(52.19)	436(47.81)		
≥10	418(10.61)	123(29.43)	295(70.57)		
Timely antiretroviral therapy				3.661	0.056
No	1,542(39.15)	888(57.59)	654(42.41)		
Yes	2,397(60.85)	1,306(54.48)	1,091(45.52)		
Initial treatment regimen				224.675	<0.001
AZT+3TC+EFV	948(24.07)	598(63.08)	350(36.92)		
AZT+3TC+NVP	355(9.01)	154(43.38)	201(56.62)		
TDF+3TC+EFV	2,237(56.79)	1,334(59.63)	903(40.37)		
D4T+3TC+NVP	91(2.31)	6(6.59)	85(93.41)		
D4T+3TC+EFV	96(2.44)	16(16.67)	80(83.33)		
Others	212(5.38)	86(40.57)	126(59.43)		
CD4 count at baseline (cells /μL)				2943.377	<0.001
≤199	1,536(38.99)	35(2.28)	1,501(97.72)		
200–349	1,538(39.05)	1,353(87.97)	185(12.03)		
350–499	564(14.32)	541(95.92)	23(4.08)		
≥500	235(5.97)	223(94.89)	12(5.11)		
Unknown	66(1.68)	42(63.64)	24(36.36)		
Recent viral load (Cope/ml)				5.692	0.017
≤999	3,837(97.41)	1,688(43.99)	2,149(56.01)		
≥1,000	102(2.59)	57(55.88)	45(44.12)		

Data are n (%). Some percentages do not sum to 100% because of missing.

^#^Data available for 3,919 subjects.

*Data available for 3,933 subjects.

AZT, zidovudine; 3TC, lamivudine; EFV, efavirenz; NVP, nevirapine; TDF, tenofovir; D4T, Stavudine.

### Clinical characteristics

Among 3,939 HIV/AIDS patients, WHO clinical stages were mainly in stage I (68.70%). There were 206 patients (5.23%) with opportunistic infections at baseline, mainly with recurrent severe bacterial infections (except pneumonia), pneumocystis pneumoniae pneumonia (PCP), and herpes zoster. The main clinical symptoms at baseline were fever (>37.5°C), skin lesions, and persistent diarrhea (adults > 1 month, children > 2 weeks). The proportion of baseline CD4 count < 200 cells/μL was 38.99%, and 200–349 cells/μL was 39.05%. There were 3,837 cases (97.41%) with the recent viral load <1,000 Cope/ml. HBsAg and anti-HCV were detected in 385 and 360 patients at baseline, respectively, of which 41 (10.65%) were HBsAg positive and 7 (1.94%) were anti-HCV positive. Details see Table 1.

### Prevalence of depressive symptoms

Of 3,939 HIV/AIDS participants, 265 (6.73%) had depressive symptoms, 158 (4.01%) had mild depressive symptoms, 65 (1.65%) had moderate depressive symptoms, 30 (0.76%) had moderate to severe depressive symptoms, and 12 (0.30%) had severe depressive symptoms. The incidence rates of depressive symptoms were 6.93% (224/3,939) in males and 5.78% (41/3,939) in females, 7.34% (161/3,939) in HIV infected patients and 5.96% (104/3,939) in AIDS. As described in Table 2.

**TABLE 2:** Analysis on the related factors for depression among HIV/AIDS in Ningbo.

	Total (*N* = 3,939)	Incidence of depression (%)	Univariate analysis		Multivariate analysis	
			χ ^2^ value	*P-value*	*OR*(95% *CI*)	*P-value*
Year of inclusion			2.853	0.583		
2,005–2,009	106	11(10.38)				
2,010–2,012	404	28(6.93)				
2,013–2,015	1,030	71(6.89)				
2,016–2,018	1,425	89(6.25)				
2,019–2,020	974	66(6.78)				
Sex			0.473	0.491		
Female	709	41(5.78)				
Male	3230	224(6.93)				
Initial age of antiretroviral therapy (years)			25.322	<0.001		
≤29	1,142	111(9.72)				
30–44	1,485	90(6.06)				
45–59	906	48(5.30)				
≥60	406	16(3.94)				
Nationality			0.064	0.801		
Ethnic minorities	95	7(7.37)				
Han nationality	3,844	258(6.71)				
Education			18.481	<0.001		
Primary and below	817	34(4.16)				
Junior high school	1,367	82(6.00)				
High school or technical secondary school	840	70(8.33)				
Junior college or above	915	79(8.63)				
Marital status^#^			29.801	<0.001		
Unmarried	1,391	135(9.71)			1.000	
Married	1,861	98(5.27)			0.675(0.501∼0.908)	0.009
Divorced or widowed	667	32(4.80)			0.571(0.380∼0.860)	0.007
Occupation*			9.572	0.048		
Workers and migrant workers	656	38(5.79)				
Housekeeping, housework, and unemployment	709	39(5.50)				
Farmers	845	48(5.68)				
Business services	749	59(7.89)				
Others	974	81(8.32)				
Household registration			9.018	0.011		
Ningbo	2,364	136(5.75)				
Other parts of Zhejiang Province	221	19(8.60)				
Other provinces	1,354	110(8.12)				
WHO clinical stages			3.227	0.358		
Phase I	2,706	194(7.17)				
Phase II	681	36(5.29)				
Phase III	331	21(6.34)				
Phase IV	221	14(6.33)				
Sexual transmission			36.764	<0.001		
Heterosexual	2,082	98(4.71)			1.000	
Homosexual	1,665	159(9.55)			1.798(1.349–2.396)	<0.001
Others	61	3(4.92)			1.044(0.321–3.404)	0.942
Unknown	131	5(3.82)			0.859(0.342–2.155)	0.745
Opportunistic infections at baseline			0.106	0.744		
No	3,733	250(6.70)				
Yes	206	15(7.28)				
Duration of antiretroviral therapy (year)			1.152	0.765		
≤3	1,247	82(6.58)				
4–6	1,362	92(6.75)				
7–9	912	58(6.36)				
≥10	418	33(7.89)				
Timely antiretroviral therapy			0.470	0.493		
No	1,542	109(7.07)				
Yes	2,397	156(6.51)				
Initial treatment regimen			0.765	0.979		
AZT+3TC+EFV	948	60(6.33)				
AZT+3TC+NVP	355	22(6.20)				
TDF+3TC+EFV	2,237	154(6.88)				
D4T+3TC+NVP	91	6(6.59)				
D4T+3TC+EFV	96	7(7.29)				
Others	212	16(7.55)				
CD4 count at baseline(cells /μL)			5.109	0.276		
≤199	1,536	89(5.79)				
200–349	1,538	109(7.09)				
350–499	5,64	46(8.16)				
≥500	2,35	18(7.66)				
Unknown	66	3(4.55)				
Recent viral load (Cope/ml)			0.556	0.456		
≤999	3,837	260(6.78)				
≥1,000	102	5(4.90)				
The type of the disease			2.943	0.086		
HIV-infected patients	2,194	161(7.34)				
AIDS	1745	104(5.96)				

Data are n or n (%). Some percentages do not sum to 100% because of missing.

^#^Data available for 3,919 subjects.

*Data available for 3,933 subjects.

AZT, zidovudine; 3TC, lamivudine; EFV, efavirenz; NVP, nevirapine; TDF, tenofovir; D4T, stavudine.

### Analysis of the related factors of depressive symptoms among HIV/AIDS

Univariate analysis showed that age, education, marital status, occupation, household registration and sexual transmission were related to depressive symptoms among HIV/AIDS. Multivariate *Logistic* regression model showed that unmarried status and homosexual transmission were significant risk factors for depressive symptoms among HIV/AIDS. Specifically, patients with married status and divorced or widowed status had about 0.675 times (95% CI = 0.501–0.908) and 0.571 times (95% CI = 0.380–0.860) greater risk for depressive symptoms compared to patients with unmarried status, the OR of depressive symptoms among HIV/AIDS with homosexual transmission was 1.793 (95% CI = 1.349–2.396) compared to those with heterosexual transmission. As shown in Table 2.

## Discussion

As a common depression screening method, both PHQ-2 scale and PHQ-9 scale had relatively good reliability and validity (23, 24). Previous studies had shown that the accuracy of combination with PHQ-2 and PHQ-9 for screening to detect major depression was more specific than using PHQ-2 alone (25), so the PHQ-2 was used as a pre-screening. In the present study, the PHQ-2 and PHQ-9 were combined to further improve the efficiency of diagnosis.

With the widespread application of antiretroviral therapy, more and more depressive symptoms occur in HIV/AIDS patients. Jiang et al. (26) found that the prevalence of depression was 18.33% among patients receiving antiretroviral therapy. The results of present study showed that the prevalence of depressive symptoms among HIV/AIDS in Ningbo was 6.73%, slightly lower than the prevalence of depressive symptoms among HIV infected patients reported in foreign cross-sectional study of 12,507 patients (8.7%) (27). Foreign studies have found that the prevalence of depressive symptoms in HIV-infected patients was approximately 20–79% (28–34), which was obviously higher than the results of this study. It could be due to differences in age, race, survey period and the tools used to define depressive symptoms. Among other HIV infected patients, the prevalence of moderate or higher depressive symptoms was over 10% (35–38), much higher than the result of present study of 2.72%.

In our study, the OR of having depressive symptoms among HIV/AIDS with married status and divorced or widowed status compared to those with unmarried status were 0.675 (95% CI = 0.501–0.908) and 0.571 (95% CI = 0.380–0.860), respectively. Unmarried HIV/AIDS patients were at greater risk of depressive symptoms, consistent with several previous studies (39–41).

The epidemics of HIV is stabilizing with the widespread use of antiretroviral therapy, HIV infection among men who have sex with men (MSM) continues to continue to increase in both developed and developing countries, with high rates of new infections especially among young MSM (42–44). In present study, participants with homosexual transmission were found to be a risk factor for depressive symptoms among HIV/AIDS compared to those with heterosexual transmission. Depressive symptoms were common among MSM with HIV infection (45, 46). It had also been reported that the incidence of depression or depressive symptoms among HIV-infected MSM in China was 43.9% (47). These results explain the conclusion of present manuscript in other perspectives. HIV-infected MSM faced further stigma and discrimination, as well as increased mental health challenges (48). Therefore, more attention should be paid to the psychological status of HIV/AIDS with homosexual transmission in Ningbo, and timely psychological intervention or treatment should be given to those patients with depressive symptoms.

The present study suffered from a few limitations. Firstly, depressive symptoms were determined by scale rather than clinical “gold standard,” and the results may be biased to some extent. In the future, it is necessary to further study the occur of depression among HIV/AIDS diagnosed by psychiatrists using a formal interview. Secondly, the cross-sectional research method cannot clear the causal relationship between the factors and depressive symptoms which needs to be further confirmed by prospective studies.

## Conclusion

This study found that marital status and sexual transmission were significantly associated with the occur of depressive symptoms among HIV/AIDS. In particular, unmarried HIV/AIDS patients were at greater risk of depressive symptoms, and heterosexual transmission had protective effects against depressive symptoms among HIV/AIDS. Therefore, scholars should pay more attention to HIV/AIDS with these characteristics described above in the future research, and timely psychological intervention or treatment should be carried out.

## Data availability statement

The datasets presented in this article are not readily available because the data used for this study is available from the corresponding author upon request. Requests to access the datasets should be directed to 2252369198@qq.com.

## Author contributions

SC analyzed the data and wrote the draft of manuscript. HH contributed to the collection of data. HH and GX generated the idea and supervised the analysis. SC, HH, and GX revised the manuscript critically. All authors put their energies into the research and approved the final version of the manuscript.
